# Supplementary material for: Effects of Exercise and Sports Intervention and the Involvement Level on the Mineral Health of Different Bone Sites in the Leg, Hip, and Spine: A Systematic Review and Meta-Analysis
Source: Int J Environ Res Public Health. 2023 Aug 7;20(15):6537. doi: 10.3390/ijerph20156537 (PMC10419061; doi:10.3390/ijerph20156537)
Supplement: Supplementary file 1 [file ijerph-20-06537-s001.zip › ijerph-2455090-supplementary.pdf]

Databases Search Strategy

Adult: 18-45 years

https://pubmed.ncbi.nlm.nih.gov/

MeSH Terms

(Bone Densities OR Density, Bone OR Bone Mineral Density OR Bone Mineral Densities OR Density, Bone Mineral OR Bone Mineral Content OR Bone Mineral Contents) AND (Sports OR Athletics OR Athletic OR Exercise OR Physical Activity OR Activities, Physical OR Activity, Physical OR Physical Activities OR Exercise, Physical OR Exercises, Physical OR Physical Exercise OR Physical Exercises OR Acute Exercise OR Acute Exercises OR Exercise, Acute OR Exercises, Acute OR Exercise, Isometric OR Exercises, Isometric OR Isometric Exercises OR Isometric Exercise OR Exercise, Aerobic OR Aerobic Exercise OR Aerobic Exercises OR Exercises, Aerobic OR Exercise Training OR Exercise Trainings OR Training, Exercise OR Trainings, Exercise) AND (Health OR Population Health OR healthies OR healthy)

https://bvsalud.org/

Example searches:

"Bone Mineral Density OR Bone Mineral Densities OR Bone Mineral Contents OR Bone Mineral Content" AND Sports$ OR Exercise$ AND Health$ OR Population Health$ OR healthies$ OR healthy$

https://www.sciencedirect.com/

Example searches:

("Bone Densities" OR "Density, Bone" OR "Bone Mineral Density" OR "Bone Mineral Densities" OR "Density, Bone Mineral" OR "Bone Mineral Content" OR "Bone Mineral Contents") AND ("Sports" OR "Athletics" OR "Athletic" OR "Exercise" OR "Physical Activity" OR "Activities, Physical" OR "Activity, Physical" OR "Physical Activities" OR "Exercise, Physical" OR "Exercises, Physical" OR "Physical Exercise" OR "Physical Exercises" OR "Acute Exercise" OR "Acute Exercises" OR "Exercise, Acute" OR "Exercises, Acute" OR "Exercise, Isometric" OR "Exercises, Isometric" OR "Isometric Exercises" OR "Isometric Exercise" OR "Exercise, Aerobic" OR "Aerobic Exercise" OR "Aerobic Exercises" OR "Exercises, Aerobic" OR "Exercise Training" OR "Exercise Trainings" OR "Training, Exercise" OR "Trainings, Exercise" AND ("Health" OR "Population Health" OR "healthies" OR "healthy")

https://pedro.org.au/

Example searches PICO:

Health* Population Health* healthy* Sport* Exercis* Physical Activity* Exercises, Physical* Aerobic Exercises* Exercise Training* Bone Mineral Density* Bone Mineral Content* Density, Bone* Bone Mineral* Bone Mineral Content* Bone Mineral Contents*

Assessment of the Methodological Quality

**Studies**	**Criterion**								**Scores**	**Adjusted ratings**
**Effect of involvement**	**1**	**2**	**3**	**4**	**8**	**9**	**10**	**11**		
Sagayama et al. [42]	1	0	0	0	1	1	1	1	4	Moderate
Bellver et al. [5]	1	0	0	0	1	1	1	1	4	Moderate
Lees et al. [39]	1	0	0	0	1	1	1	1	4	Moderate
Tam et al. [44]	1	0	0	0	1	1	1	1	4	Moderate
Hind et al. [22]	1	0	0	0	1	1	1	1	4	Moderate
Piasecki et al. [10]	1	0	0	1	1	1	1	1	5	Good
Bolam et al. [41]	1	0	0	1	1	1	1	1	5	Good
**Effect of training intervention**										
Caruso et al. [55]	1	0	0	1	1	1	1	1	5	Good
Mosti et al. [56]	1	0	0	0	1	1	1	1	4	Moderate
Suarez-Arrones et al. [62]	1	0	0	1	1	1	1	1	5	Good
Feito et al. [57]	1	0	0	0	1	1	1	1	4	Moderate
Kurgan et al. [50]	1	0	0	1	1	1	1	1	5	Good
Infantino et al. [51]	1	0	0	0	1	1	1	1	4	Moderate
